# Human Leukocyte Antigen Haplotypes Predisposing to Celiac Disease in Patients With Endometriosis

**DOI:** 10.1111/aji.70079

**Published:** 2025-04-22

**Authors:** Silvia Vannuccini, Virginia Manzi, Mirko Tarocchi, Nico Donati, Francesco La Torre, Federico Toscano, Antonino Salvatore Calabrò, Felice Petraglia

**Affiliations:** ^1^ Department of Experimental, Clinical and Biomedical Sciences “Mario Serio” University of Florence Florence Italy; ^2^ Department of Maternal and Child Health Careggi University Hospital Florence Italy; ^3^ Clinical Gastroenterology Unit Careggi University Hospital Florence Italy

**Keywords:** celiac disease, endometriosis, genetic predisposition, HLA DQ2, HLA DQ8, human leukocyte antigen

## Abstract

**Problem:**

Immunological abnormalities are well recognized in the pathogenesis of endometriosis and the co‐existence of endometriosis with inflammatory bowel disease (IBD) and celiac disease (CD), along with other systemic immune disorders, is clinically relevant. Recent genetic studies revealed some shared genetic traits associated with the co‐occurrence of endometriosis with different gastrointestinal or autoimmune disorders, highlighting common biological pathways. Since class II human leukocyte antigen (HLA) genes, HLA‐DQ2 and ‐DQ8, show the strongest and best‐characterized genetic susceptibility for CD, the present study aims to explore the presence of these haplotypes in non‐celiac patients with endometriosis.

**Method of Study:**

A group of patients with endometriosis (*n* = 126) participated in the study and were compared to healthy women (*n* = 379), as controls. Subjects who were diagnosed with CD or who tested positive for CD antibodies were excluded. All patients and controls were genotyped for HLA haplotypes predisposing to CD (DQ2, DQ8). In the group of endometriosis patients who tested positive for DQ2 and/or DQ8, symptoms were also investigated.

**Results:**

At least one of the HLA‐DQ2 and ‐DQ8 genotypes was detected in 43.3% of non‐celiac endometriosis patients (OR: 1.82, 95% CI: 1.11–2.81), whereas 29.5% (*p* < 0.01) of healthy women presented HLA haplotypes predisposing to CD. In endometriosis patients, no significant difference was shown between positive and negative in terms of endometriosis phenotype, or gynecological, and non‐gynecological symptoms.

**Conclusions:**

Our data revealed a significantly greater prevalence of predisposing haplotypes for CD in non‐celiac patients with endometriosisthan in healthy subjects, suggesting that a common genetic background may explain the co‐occurrence of endometriosis and CD.

## 1 Introduction

Endometriosis presents an endocrine and inflammatory pathogenesis, with immunological abnormalities, including alterations in the cell‐mediated and humoral response [1, 2, 3, 4]. Immune mechanisms are also crucial for celiac disease (CD), a chronic enteropathy initiated by exposure to dietary gluten in genetically predisposed individuals [5]. It is characterized by specific autoantibodies against tissue transglutaminase 2, endomysium, and/or deamidated gliadin peptide [6]. The percentage of celiac individual carrying the human leukocyte antigen (HLA)‐DQ2 haplotype is 90%, whereas approximately 5% express the ‐DQ8 haplotype, and almost 5% of the remaining subjects carry at least one of the two alleles (i.e., beta chain *02 or alpha chain *05) [7, 8]. The prevalence of these haplotypes is around 25%–40% in the general population, but only 2%–3% of carriers would develop CD, supporting the concept that is necessary but not sufficient for the development of CD [9].

Interestingly, epidemiological studies have shown an association between endometriosis and CD [10, 11], along with irritable bowel syndrome (IBS) [12], inflammatory bowel disease (IBD) [13], and various autoimmune diseases [14]. A clear analysis of cause‐effect associations is not possible, even though patients with CD have a higher risk of developing endometriosis within the first year after diagnosis [15]. Furthermore, the association between endometriosis and gastrointestinal disorders is supported by a genetic background with shared risk loci, highlighting common biological pathways [16].

The present study aimed to explore the presence of HLA haplotypes predisposing to CD in non‐celiac patients with endometriosis, in order to evaluate a possible common genetic background.

## 2 Materials and Methods

An observational prospective study was conducted on a group of patients referred to our Endometriosis Center (*n* = 126) between 2021 and 2023. The diagnosis of endometriosis was based either on a previous surgical and histological confirmation or on the combination of clinical symptoms and imaging (transvaginal ultrasound (TVUS) and/or magnetic resonance (MRI)) [17, 18, 19]. Three major phenotypes were distinguished according to the localization of lesions: ovarian endometriosis, peritoneal superficial and deep infiltrating endometriosis, or mixed phenotypes [20]. The presence of associated adenomyosis was also reported (*n* = 82; 65%). The presence of endometriosis‐associated pain symptoms (dysmenorrhea, dyspareunia, non‐menstrual pelvic pain, dysuria, dyschezia) and non‐gynecological symptoms (constipation, diarrhea, alternating bowel habits, abdominal bloating, headache, migraine, back pain) was assessed. Pelvic pain scores and associated symptoms were assessed using a visual analog scale (VAS); if VAS ≥ 7, pain was defined as severe. Among patients with endometriosis, those with a diagnosis of CD, which was already made or indicated by positivity for anti‐endomysium, anti‐transglutaminase, or anti‐gliadin antibodies at screening, were excluded from the analysis.

The study population was compared to a control group of healthy female subjects scheduled for bone marrow donation, who were undergoing a comprehensive diagnostic evaluation, including HLA haplotyping [21]. Bone marrow donors had a negative medical history and medical exams within the normal range. They were also screened for antibodies associated with CD and tested negative to be cleared to donate. The control group consisted of 379 female subjects. Age, body mass index (BMI), and ethnicity, retrieved from electronic medical records, were not significantly different between cases and controls.

### 2.1 Sample Collection and Analysis

The patients were assessed in terms of HLA genotype, by determining the presence of DQ2 or DQ8 after DNA extraction from peripheral blood samples using the salting‐out technique, followed by PCR‐SSP analysis (Polymerase Chain Reaction‐Sequence Specific Primers).

The HLA class II locus DQ, located on chromosome 6p21, is the most clinically relevant genetic factor associated with CD. HLA‐DQ2.5 is encoded by the DQA1*05:01 (or DQA1*05:05) and DQB1*02:01 (or DQB1*02:02) genes, whereas DQ2.2 is encoded by HLA‐DQA1*02:01 and HLA‐DQB1*02:02. DQ8 comprises DQA1*03:01 and DQB1*03:02 [9].

Anti‐deamidated gliadin antibodies (DGP) were tested using the ELISA technique with the deaminated peptide method (α‐Glia‐PEP, Eurospital, Trieste, Italy). The reference values for this method were established at 5.5 U/mL for IgA AGA and 10 U/mL for IgG AGA. Anti‐endomysial antibodies (EMA) were assessed using the Indirect Immunofluorescence technique on monkey esophagus sections (Eurospital). Lastly, the ELISA technique was used to test for anti‐transglutaminase antibodies (t‐TG) with a commercially available kit that utilizes recombinant transglutaminase as the antigen (EuTG, Eurospital).

### 2.2 Statistical Analysis

The collected data were entered into a database and analyzed. Percentages, means, and standard deviations (SD) have been extrapolated, followed by the creation of charts and tables for descriptive analysis. Unpaired Student's *t*‐test was used to compare continuous parametric variables and the Mann–Whitney *U* test was used for non‐parametric variables between the groups. *χ*2 test (or Fisher's exact test if necessary) and Odds Ratio calculation were performed to compare HLA DQ2 and DQ8 positivity in cases and controls, and according to referred symptoms. The level of significance was set at *p* < 0.05.

## 3 Results and Discussion

The final study population included 113 patients with endometriosis, after 13 patients (10.3%) were excluded because of positive results for celiac autoantibodies at screening or a previous diagnosis of CD, confirming a higher prevalence of CD in women with endometriosis than in controls (10.3% vs. 1.26%, *p* < 0.001) [14]. Similarly, individuals with CD have a greater risk of developing endometriosis, especially within the first year after diagnosis [15], making both endometriosis and CD patients at mutual higher risk of disease development. Several immune‐mediated diseases share susceptibility loci with both endometriosis and CD, shedding light on the potential reasons for co‐occurrence between these diseases [14, 22, 23].

**FIGURE 1:**
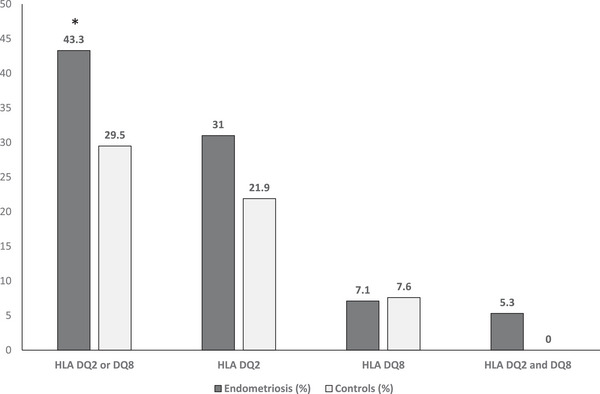
Bar chart showing HLA DQ2 and HLA DQ8 distribution in endometriosis patients and control group, as percentages of the total population. *p < 0.01.

In our study, genetic analysis revealed that in the endometriosis group without evidence of CD, 49 patients (43.3%) tested positive for HLA DQ2 or DQ8, which was significantly more frequently than in the control group (29.5%) (*p* = 0.006) (OR: 1.82, 95% CI: 1.11–2.81). Specifically, among patients with endometriosis resulted positive, 35/49 (71.4%) carried HLA DQ2, 8/49 (16.3%) were carriers of HLA DQ8, and 6/49 (12.2%) were positive for both HLA DQ2 and DQ8. In the control group, 83/379 women (21.9%) carried DQ2 and 29/379 (7.6%) carried DQ8 (Figure 1). In contrast, the association between endometriosis localization and positivity for HLA‐DQ2 or HLA‐DQ8 haplotypes was not statistically significant.

This is the first report showing a significantly higher presence of these haplotypes in non‐celiac endometriosis patients. HLA‐C and HLA‐G were previously investigated in endometriosis [24, 25]. However, only a retrospective study has been published specifically on HLA‐DQ2 and DQ8, showing, unlike our results, a similar prevalence of these haplotypes in cases and gynecological controls, even though some methodological limitations should be acknowledged [26].

It is well‐known that the presence of the human leukocyte antigens HLA‐DQ2 and HLA‐DQ8 haplotypes is a necessary, although not sufficient, condition to develop CD [27], even though it remains one of the main risk factors for CD. In non‐celiac disease patients, early disruption of the gut barrier and subsequent increased intestinal permeability may precede gluten‐induced immune events. This sequence of events may involve intestinal dysbiosis. For instance, infants carrying the HLA‐DQ2 haplotype exhibit altered early gut microbiota composition [28], with higher proportions of *Firmicutes* and *Proteobacteria* and lower proportions of *Actinobacteria* than those with lower genetic risk. The HLA‐DQ2 genotype plays a significant role in shaping the gut microbiota, particularly in individuals at risk for autoimmune diseases such as CD and type 1 diabetes. The genotype influences the abundance and diversity of specific bacterial groups, which may contribute to disease development. Furthermore, in genetically susceptible individuals, CD can be triggered by environmental factors, including viral infections and microbiota imbalances [29]. The gut microbiome plays a crucial role in immune system regulation, and emerging evidence suggests that its alterations may contribute to chronic immune disorders, including CD. Some bacteria mimic gliadin epitopes, potentially enhancing immune responses, whereas others may interact with gluten to exacerbate intestinal inflammation. The microbiome also influences inflammation through the production of pro‐ and anti‐inflammatory cytokines and metabolites [30, 31]. These findings suggest that specific disease‐biased host genotypes may influence initial gut colonization and contribute to disease risk, as well as in gynecological function and pregnancy [21]. Recently, genomics research has shown that the human microbiota is significanlty related to female reproductive health. In particular, the gut microbiota is associated with many inflammatory diseases, including endometriosis [32]. In particular, genes involved in the immune response and the regulation of pro‐inflammatory cytokines appear to play a key role in the development of both CD and endometriosis. The impairment of the intestinal barrier and alterations in the microbiota could also represent a mechanism through which the interaction between genetic predisposition and environmental factors contributes to their shared pathogenesis. The mechanism by which the gut microbiota affects endometriosis may involve estrogen, immunity, and inflammation [33, 34], even though the specific inter‐relationship is yet to be accurately defined. However, the pathogenetic and clinical significance of DQ2 and DQ8 haplotypes in non‐celiac endometriosis patients is yet to be fully elucidated, although DQ8 and to a lesser extent DQ2 are also associated with the occurrence of some autoimmune diseases such as rheumatoid arthritis, which are notably more common in endometriosis patients [14].

Endometriosis patients positive for the DQ8 or DQ2 haplotypes showed the following symptoms: abdominal bloating (57.1%), migraine (12.2%), headache (40.8%), back pain (40.8%), diarrhea or constipation (10.2%), and alternating bowel habits (28.6%), with no significant difference compared to those who tested negative (Table 1). No statistically significant difference was also noted in terms of gynecological symptoms. The symptom distribution in non‐celiac patients positive for HLA‐DQ2 or DQ8 may be varied and non‐specific. In nonceliac gluten sensitivity, the prevalence ranges from 25% to 50% [35]. This condition refers to the presence of intestinal or extraintestinal signs or symptoms, or both, resulting from the ingestion of gluten‐containing grains with symptomatic improvement upon their withdrawal [36]. The frequent occurrence of these intestinal or extra‐intestinal symptoms, such as abdominal bloating, decreased/increased passage of stool, headache, and fatigue, is also reported in endometriosis patients, but evidence on food allergy and/or intolerance to gluten in affected women is still lacking [37].

**TABLE 1:** Endometriosis group characteristics and symptoms according to HLA haplotype.

Baseline characteristics	HLA DQ2 or DQ8 positive (*n* = 49)	HLA DQ2 or DQ8 negative (*n* = 64)	*p*
Body mass index (kg/m^2^)	21.6 ± 2.7	21.5 ± 3.2	0.860
Age (years)	32.2 ± 6.6	32.2 ± 7.8	0.319
Caucasian ethnicity	43 (87.7)	55 (86.0)	0.777
Endometriosis phenotype			
*Ovarian endometriosis*	10 (20.4)	19 (29.7)	0.263
*Peritoneal superficial*	10 (20.4)	16 (25.0)	0.565
*Deep infiltrating endometriosis*	35 (71.4)	48 (75.0)	0.670
*Mixed*	11 (22.4)	17 (26.6)	0.615
Associated adenomyosis	36 (73.5)	46 (71.9)	0.850
**Symptoms**			
Abdominal bloating	28 (57.1)	43 (67.2)	0.273
Migraine	6 (12.2)	12 (18.8)	0.349
Headache	20 (40.8)	17 (26.6)	0.109
Back pain	20 (40.8)	36 (56.3)	0.103
Constipation	5 (10.2)	11 (17.2)	0.291
Diarrhea	5 (10.2)	12 (18.8)	0.207
Alternating bowel habits	14 (28.6)	11 (17.2)	0.148
Dysmenorrhea	45 (91.8)	59 (92.2)	0.945
Severe dysmenorrhea	34 (69.4)	35 (54.7)	0.112
Chronic pelvic pain	27 (55.1)	42 (65.6)	0.255
Dyspareunia	34 (69.4)	48 (75.0)	0.507
Dyschezia	6 (12.2)	9 (14.1)	0.777
Dysuria	15 (30.6)	24 (37.5)	0.445

*Note:* Continuous variables are expressed as mean ± SD and binomial variable are expressed as *n* (%).

Evidence suggests that the diet of patients with endometriosis may play a key role in improving associated gastrointestinal symptoms. It remains uncertain which dietary approach could effectively ease endometriosis‐related pain; evidence suggests that the most significant perceived reductions in symptoms occur when gluten, dairy, or soy are removed from the diet, as well as when vegetables are added [38]. However, currently there are no strong evidence to support a recommendation to follow a gluten‐free diet only because of an endometriosis diagnosis [39].

A limitation to acknowledge is the potential selection bias due to the choice of bone marrow donors as controls, considering the extensive diagnostic protocol they underwent, thus resulting in a highly selected population. However, the prevalence of HLA‐DQ2 and DQ8 was comparable to that of the general population [9].

In conclusion, the occurrence of DQ2 and DQ8 haplotypes among non‐celiac women with endometriosis is notably elevated compared with that of healthy subjects, indicating that endometriosis and CD share a common genetic trait. This intersection, along with the observation of a significant association between endometriosis and overt CD, may have practical implications for patient care, suggesting a multidisciplinary approach to women suffering from endometriosis. Specifically, a gastroenterology follow‐up for those endometriosis patients carrying haplotypes predisposing for CD may be suggested.

## Ethics Statement

All the procedures performed in the studies that involved human participants were in accordance with the ethical standards of the Institutional and National Research Committee, and also with the 1964 Declaration of Helsinki and its later amendments. Approval from the institutional review board: protocol no. 13742.

## Conflicts of Interest

All authors declare no conflict of interest.

## Data Availability

The data that support the findings of this study are available on request from the corresponding author. The data are not publicly available due to privacy or ethical restrictions.
